# Trophic Discrimination Factors of Stable Carbon and Nitrogen Isotopes in Hair of Corn Fed Wild Boar

**DOI:** 10.1371/journal.pone.0125042

**Published:** 2015-04-27

**Authors:** Michaela Holá, Miloš Ježek, Tomáš Kušta, Michaela Košatová

**Affiliations:** 1 Department of Game Management and Wildlife Biology, Faculty of Forestry and Wood Sciences, Czech University of Life Sciences Prague, Prague, Czech Republic; 2 Department of Ecology, Faculty of Environmental Sciences, Czech University of Life Sciences Prague, Prague, Czech Republic; University of Missouri Kansas City, UNITED STATES

## Abstract

Stable isotope measurements are increasingly being used to gain insights into the nutritional ecology of many wildlife species and their role in ecosystem structure and function. Such studies require estimations of trophic discrimination factors (i.e. differences in the isotopic ratio between the consumer and its diet). Although trophic discrimination factors are tissue- and species- specific, researchers often rely on generalized, and fixed trophic discrimination factors that have not been experimentally derived. In this experimental study, captive wild boar (*Sus scrofa*) were fed a controlled diet of corn (*Zea mays*), a popular and increasingly dominant food source for wild boar in the Czech Republic and elsewhere in Europe, and trophic discrimination factors for stable carbon (Δ^13^C) and nitrogen (Δ^15^N) isotopes were determined from hair samples. The mean Δ^13^C and Δ^15^N in wild boar hair were –2.3 ‰ and +3.5 ‰, respectively. Also, in order to facilitate future derivations of isotopic measurements along wild boar hair, we calculated the average hair growth rate to be 1.1 mm d^-1^. Our results serve as a baseline for interpreting isotopic patterns of free-ranging wild boar in current European agricultural landscapes. However, future research is needed in order to provide a broader understanding of the processes underlying the variation in trophic discrimination factors of carbon and nitrogen across of variety of diet types.

## Introduction

Based on the adage that “you are what you eat”, researchers have widely used stable carbon and nitrogen isotope analysis of animal tissues to address a wide variety of topics related to wildlife management, including trophic interactions, animal movement and migration, and diet composition and habitat use in many species (e.g. [1–4]). The stable isotope ratios of carbon (^13^C/^12^C, reported as δ^13^C) and nitrogen (^15^N/^14^N, reported as δ^15^N) in animal tissues reflect the isotopic signature of diet and can be used to determine the relative contributions of isotopically distinct food sources to an animal’s diet [5–8]. Dietary inferences based on plant foliar δ^13^C values generally rely on divergent photosynthetic pathways of C_3_ (Calvin-Benson photosynthetic cycle [9]) and C_4_ (Hatch-Slack cycle [10]) plants. Most C4 plants have δ^13^C values between—16 and—9 ‰ with an average of—13.1 ± 1.2 ‰, whereas most C3 plants range from—35 to—22 ‰ with an average of—27.1 ± 2.0 ‰ [11, 12]. In contrast, δ^15^N values of vegetation integrate terrestrial nitrogen cycling and can vary greatly due to a number of abiotic [13] and physiological [14] factors.

The measurements of δ^13^C and δ^15^N in animal tissues yield time-integrated information of assimilated and not just ingested diet, thereby avoiding limitations often associated with more traditional methods of dietary reconstruction such as fecal or stomach content analyses [15, 16]. The period of integration of diet-derived isotopic signature depends on tissue-specific metabolism [15], and therefore, different tissues provide records of animals’ dietary histories integrated over different time periods [15, 17]. Tissues with higher metabolic activity (e.g. blood plasma, liver) reflect recent dietary intake, whereas tissues with lower metabolism (e.g. red blood cells, bone collagen) reflect diet integrated over longer time-periods [15, 17]. Metabolically inert tissues, such as hair, reflect the diet consumed during the period of their growth and store this information in a chronological manner [18]. Every growth section of the hair reflects the isotopic information of diet consumed during the time period when it was produced. Therefore, a sequential analysis of hair (i.e. hair is sectioned into sequential segments so temporal isotopic variation along hair length unit can be analyzed) allows us to assess the recent feeding history of an animal, whereas serially collected hairs (i.e. hairs collected at different time periods) provide information on the long-term dietary intake (e.g. [19–21]). However, conversion of the isotope data along the length of hair into a temporal record requires estimates of the average hair growth rate of the species under study [18]. Moreover, hair tissues can be obtained non-invasively and with minimal disturbance to an animal and can be easily preserved and stored.

The isotopic ratios of diet sources are not transmitted directly to a consumer’s tissue, since there is a systematic difference between the isotopic composition of the consumer tissues and that of the diet, i.e. the trophic discrimination factor (TDF). The values of TDFs need to be taken into account while reconstructing diet of animals by stable isotope analysis [15, 22–24]. The TDF is typically expressed as Δ^13^C for C and Δ^15^N for N, where Δ represents the difference in isotopic composition between the diet and an animal’s tissue [22]. These TDFs may vary depending upon many factors, including the studied species, trophic level, type of tissue analyzed, growth rate, differential digestibility, and diet quality (e.g. [25–28]). Values of TDFs may thus be unique for species, tissue and diet [25, 27]. However, dietary inferences based on stable isotope analysis of tissues of wild animals are often unreliable, because researchers rely on generalized, fixed, and not experimentally derived TDFs from the literature without taking into account the species under study, tissue analyzed, or the type of diet [27, 29, 30]. The use of inaccurate TDFs can lead to possible errors and misinterpretation of field data [30, 31]. Therefore, an experimental determination of species- and tissue- specific discrimination factors under controlled conditions is essential and may greatly improve our understanding of the mechanisms underlying the variation in TDFs [27, 30].

Despite the rapid progression of stable isotope analysis in mammalian research, the TDFs of stable carbon and nitrogen isotopes in various tissues have been determined only for a limited number of mammalian omnivores [17, 32–36]. Regarding suids, to our knowledge, there are only two studies on TDFs in multiple tissues of domestic pig [37, 38], and none for wild boar (*Sus scrofa*).

The wild boar is an opportunistic omnivore with a wide diet breadth. Although wild boar primarily consume plant matter (80–90% of total food mass), they may also consume small vertebrates and invertebrates, and occasionally inorganic material [39, 40]. On the other hand, some studies have shown that, when available, wild boar prefer to feed almost exclusively on agricultural crops (e.g. [41, 42]. Specifically, corn (*Zea mays*) is an important and dominant diet component for wild boar in the Czech Republic and elsewhere in Europe [39, 41]. The increased cultivation of corn in recent years due to the bio-energy production as well as its use in supplementary feeding, have rendered it extremely important yet an easy food source for wild boar, constituting about 87% of their diet year-round [41]. In most of central Europe, corn is available in fields for free-ranging wild boar from June-July until the harvest in November-December. Wild boar are frequently observed visiting corn fields during the entire cultivation period (i.e. August-November) [43]. Wild boar populations have been rapidly increasing in numbers and distribution throughout Europe during recent decades [44] resulting in growing conflicts with farmers due to substantial losses of agricultural crops [39, 41]. Therefore, the feeding ecology, particularly in the context of diet selection of wild boar, is of particular interest, especially in relation to corn.

Here we performed an experimental study in order to establish baseline information for interpreting stable-isotope patterns in free-ranging wild boar thriving in European agricultural landscapes. We determined stable carbon and nitrogen isotope discrimination factors in hair samples of four 3-year old captive wild boar (*Sus scrofa*) fed with a controlled diet of corn (*Zea mays*) over a 4-month period. Furthermore, in order to facilitate future derivations of the temporal dietary record from isotopic signatures along hair length units, we calculated the average growth rate (mm/day) of wild boar hair.

## Materials and Methods

### Ethics statement

This study was carried out in accordance with the recommendations in the Guide for Care and Use of Animals of the Czech University of Life Sciences Prague. The protocol was approved by the Animal Care and Use Committee of the Czech Ministry of the Environment (Permit number: 15106/ENV/14-825/630/14). All care was undertaken to minimize stress and suffering to the animals.

### Study design

We conducted our feeding experiment from June to October 2013 at the Sedlice game park (49°21.705´, E13°59.343´), Czech Republic. This time period was selected as it coincides with the availability of corn in fields for free-ranging wild boar. Four 3-year old male wild boar were fed with corn grain (*Zea mays*, crude protein = 8.68%, carbon content = 40.72%) for the duration of the experiment (4 months). The corn grain was obtained from a single batch to minimize isotopic heterogeneity. The captive wild boar were held in an experimental outdoor enclosure (0.5 ha) over the study period. Feed and water were provided *ad libitum*. A 4-month period was selected as it has been shown to be sufficient to allow hair to reach an isotopic equilibrium of δ^13^C and δ^15^N with diet [32, 45, 46].

### Sample collection

At the start of the experiment (day 0), when the corn grain was provided, two patches (c. 6 cm^2^) of hair at two different body parts from each individual (i.e. shoulder and rump) were shaved to the skin. These hair samples were not analyzed for the isotopic signatures since they reflected only the diet prior to the experiment. At the first sampling period (day 42), we plucked a bundle of hair (including hair root) from the previously shaved patches from each wild boar. At the end of the experiment (day 140), we plucked a bundle of hair again within the same patches where the hair were sampled during the first sampling period earlier, but now at different location within the same patch. Thus, the hair samples collected at the end of the experiment reflected the dietary information from day 0. Since the wild boar hairs were shaved at the beginning of the experiment, we assumed that their isotopic signatures would reflect only the research diet when collected. Only guard hairs were selected from each animal. The corn grain samples were collected monthly.

### Hair growth rate

All hair samples collected from individual wild boar at the first sampling period (day 42) were measured and the mean length (mm) of the shoulder and rump hair was determined. The rate of growth for shoulder and rump hair of each individual was calculated by dividing the mean length of the shoulder/rump hair by the number of days since the beginning of the experiment (i.e. 42 days).

Since the differences in hair growth rate between individual body parts were minimal, we took a mean growth rate in this study. The study individuals were on a constant diet over the duration of the experiment and accounting for the slight observed difference would not affect the interpretation of our results. Hence, for all calculations, we used the average growth rate of wild boar hair (i.e. the mean length of both shoulder and rump hair (46.48 mm) divided by 42 d.).

### Sample preparation and stable isotope analyses

**Fig 1:**
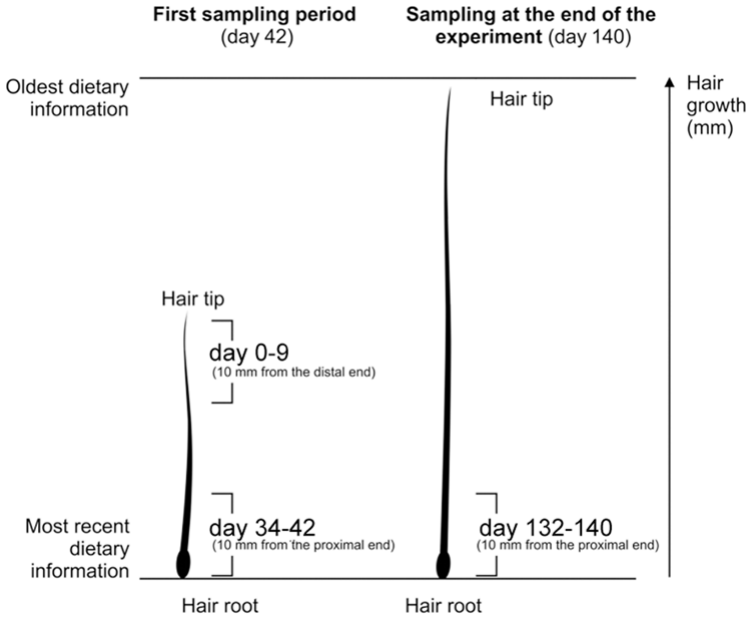
Sequences of wild boar hair used for isotope analysis of δ^13^C and δ^15^N. The wild boar hair were collected at the first sampling period (day 42) and at the end of the experiment (day 140) from the shoulder and rump of each wild boar.

To compare the degree of incorporation of δ^13^C and δ^15^N values of the corn grain into hair at different periods of the experiment, we cut 10 mm sections from the distal end of hair and 10 mm sections from the proximal end (hair root included) of the hair samples collected at the first sampling period (day 42) and only these sections were used for isotope analysis. Based on the average hair growth rate of wild boar calculated in the previous step (i.e. 1.1 mm/day), the 10 mm sections from the distal ends of the hair represent the information on the first c. 9 days from the start of the experiment (i.e. day 0–9) and the 10 mm sections from the proximal ends reflect the most recent information prior to the first sampling period (i.e. day c. 34–42). In order to obtain the most recent dietary information prior to the end of the experiment, we cut 10 mm sections from the proximal end (hair root included) of the hair collected at the end of the experiment (day 140). Therefore, we obtained dietary information on the last c. 9 days of the experiment (i.e. 132–140; Fig 1). We visually inspected roots of the plucked hair and only hairs in the anagen growth phase with undamaged roots were used for isotope analysis [47].

Individual hair samples were washed in 0.25M sodium hydroxide solution to remove any contaminants and oil residues followed by two separate washes in purified water. The washed hair samples were placed in sanitized screw top vials and dried overnight at 60°C. The corn grain samples were ground to a fine powder, homogenized, and dried overnight at 60°C.

The δ^13^C and δ^15^N values of all samples were determined using EA-IRMS (Elemental Analysis-Isotope Ratio Mass Spectrometry) coupled with Europa Scientific 20–20 IRMS at Iso-Analytical Ltd. (Cheshire, UK). The results of isotope analyses are presented as δ^13^C (‰) relative to the Vienna PeeDee Belemnite (V-PDB) standard and δ^15^N (‰) relative to nitrogen in air and were calculated as follows: $\delta X=\left(\frac{R_{sample}}{R_{standard}}-1\right)*1000,\;$where *δX* is δ^13^C or δ^15^N, and *R* is the respective ^13^C/^12^C or ^15^N/^14^N ratio. The internal standards, IA-R042 (powdered bovine liver, δ^13^C_V-PDB_ = –21.6 ‰, δ^15^N_AIR_ = 7.6 ‰) for hair samples and IA-R001 (wheat flour, δ^13^C_V-PDB_ = –26.4 ‰, δ^15^N_AIR_ = 2.5 ‰) for corn samples, were used as reference material to ensure the analytical precision of the measurements. IA-R042, a mixture of IA-R005 and IA-R045 and a mixture of IA-R006 and IA-R046 were analyzed for quality control of the hair samples. IA-R001, a mixture of IA-R005 and IA-R045 and a mixture of IA-R006 and IA-R046 were analyzed for quality control of the corn samples. These working standards were then calibrated against IAEA standards N1 and CH6. The precision of measurements was better than 0.1 ‰ (one standard deviation) for both elements.

### Trophic discrimination factor

We calculated the hair-diet discrimination factors of carbon (Δ^13^C) and nitrogen (Δ^15^N) by subtracting the mean δ^13^C and δ^15^N values of the corn grain from the mean δ^13^C and δ^15^N values of the sections of the hair samples collected at the end of the experiment (day 140).

### Statistical analysis

We evaluated possible differences in δ^13^C and δ^15^N values in hair samples collected at the first sampling period (day 0–9, 34–42) and those taken at the end of the experiment (day 132–140) using Paired Student´s t-tests. Mixed-effects models with repeated measures were not performed, because running a model with fixed and random effects and such a small sample size (here n = 4) would result in over parameterizing the models [48]. Similar experimental studies focusing on TDFs in the past have also avoided mixed-effects models supposedly for the same reason as we state (e.g. [49, 50, 51]). Measurements of mean δ^13^C and δ^15^N values are reported as means ± SD. Significance was tested at α = 0.05 level. All statistical analyses were performed using R software, version 3.0.3 [52].

## Results

The mean lengths and rates of growth of the shoulder and rump hair of individual wild boar are shown in Table 1.

**Table 1:** The mean length and rate of growth of the shoulder and rump hair of individual wild boar.

	Shoulder		Rump	
	Mean length (mm)	Growth rate (mm d^-1^)	Mean length (mm)	Growth rate (mm d^-1^)
Individual **1**	47.57 ± 2.21	1.13	43.02 ± 3.46	1.02
Individual **2**	45.38 ± 4.77	1.08	42.38 ± 3.92	1.04
Individual **3**	49.98 ± 3.09	1.19	49.52 ± 4.17	1.17
Individual **4**	48.58 ± 3.04	1.15	46.42 ± 4.21	1.1
**Mean ± SD**	47.88 ± 1.94	1.13 ± 0.05	45.33 ± 3.30	1.02 ± 0.07

Values are reported as mean ± SD; n = 100 for individuals 1, 2, and 3 (i.e. n = 50 for shoulder and n = 50 for rump); n = 40 for individual 4 (i.e. n = 20 for shoulder and n = 20 for rump).

On average, wild boar hair grew 46.48 mm in 42 days, thus yielding an average growth rate of 1.1 mm d^-1^. Therefore, the 10 mm section of wild boar hair reflected the diet consumed during c. 9 days of hair growth.

The mean δ^13^C and δ^15^N values of the corn grain and of the hair samples collected throughout the duration of the experiment, as well as the mean TDFs for carbon and nitrogen in wild boar hair are shown in Table 2. TDFs for δ^13^C and δ^15^N in wild boar hair were—2.3 ‰ and +3.5 ‰, respectively, indicating a depletion in ^13^C relative to the corn grain values, and enrichment in ^15^N.

**Table 2:** Mean carbon (δ^13^C) and nitrogen (δ^15^N) isotope values of the corn samples (n = 4), and the hair samples collected at the first sampling period (day 0–9, day 34–42) and at the end of the experiment (day 132–140), and mean trophic discrimination factors (TDF) in wild boar (*Sus scrofa*) hair.

	Corn grain	Hair (day 0–9)	Hair (day 34–42)	Hair (day 132–140)	TDF
**Carbon**	–12.0 ± 0.03	–18.8 ± 0.7	– 14.3 ± 0.4	–14.3 ± 0.2	–2.3
**Nitrogen**	4.2 ± 0.2	7.8 ± 0.3	7.7 ± 0.1	7.7 ± 0.2	3.5

Values are reported as mean ± SD. The Δ^13^C and Δ^15^N values were calculated by subtracting the mean carbon or nitrogen isotope values of the hair samples collected at the end of the experiment and mean carbon or nitrogen isotope value of the corn grain. All values of δ^13^C and δ^15^N are presented in ‰.

The mean values of δ^13^C in the hair samples representing days 0–9 were significantly different from those collected at the end of the experiment (day 132–140; t = -16.28, n = 8, p < 0.0001). In contrast, the mean values of δ^15^N did not differ between the days 0–9 and 132–140 (t = 0.832, n = 8, p > 0.583). There were no differences between the days 34–42 and 132–140 for either δ^13^C or δ^15^N values in hair (δ^13^C: t = -0.395, n = 8, p > 0.704; δ^15^N: t = 0.566, n = 8, p > 0.5888).

## Discussion

Our study provides the first experimentally derived trophic discrimination factors of stable carbon (Δ^13^C) and nitrogen isotopes (Δ^15^N) and the average growth rate reported for hair of wild boar (*Sus scrofa*) fed with a controlled diet of corn (*Zea mays*).

Reliable dietary inferences based on isotopic data of serially sampled hair require conversion of spatial isotopic records into temporal records. Therefore, estimates of hair growth rate are essential to increase the degree of accuracy of such conversions [18]. We estimated the average growth rate of wild boar hair to be 1.1 mm d^-1^based on the average of the growth rates of shoulder and rump hair. The growth rates from these two body locations were only slightly different (i.e. 1.13 mm d^-1^ for shoulder hair and 1.02 mm d^-1^ for rump hair). We analyzed 10 mm sections of both shoulder and rump hair and if we were to apply the body part- specific growth rates separately, the 10 mm sections of shoulder hair would reflect 9 days of the experiment and the 10 mm sections of rump hair would reflect 10 days of the experiment. Moreover, since our study animals were on a constant diet over the duration of the experiment, we believe that this 1 day difference would not significantly affect the interpretation of our results. As a cautionary note, we suggest that researchers should consistently use the same sampling locations on bodies of their study individuals when estimating diets by stable isotope analysis of hair. Overall, in studies involving reconstructing diets from isotopic signatures, one always encounters scenarios where a trade-off between analytical precision, spatial (and thus also temporal) resolution, and effort and cost must be made, and therefore some of the assumptions may be compromised [18].

Our values for hair growth rate were derived using recently shaved hair, however previous studies have shown that there is no change in growth rate associated with shaving [53, 54]. Some studies report that nutrition, hormonal status, or environmental conditions may also influence rate of hair growth [55, 56, 57]. Our study animals were fed with a single item diet of a low nutritional quality and we realize that estimates of hair growth rate may differ for free-ranging animals consuming diverse diets of moderate- or high- quality, providing an opportunity for further investigation. The timing of our experiment (June-October) was selected as it matches with the availability of corn for free-ranging animals in Central Europe (i.e. June/July-November/December) and previous studies have shown that, when available, corn represents a major component of wild boar diet, constituting about 87% of biomass intake [41, 42]. Consequently, we believe that our estimated hair growth rates for captive wild boar would reflect growth rates of free-ranging individuals thriving in agricultural areas. These rates should hence be applicable in areas with high acreage of corn fields. Altogether, in order to reliably and accurately interpret isotopic signatures of hair, the species- (body part-) specific hair growth rates estimations are required and we hereby provide the first such estimates for wild boar, although within restricted conditions.

An important consideration for the successful interpretation of isotopic signatures of hair is the determination of the trophic discrimination factors [27, 30]. Our calculations of the mean Δ^13^C in wild boar hair (– 2.3 ‰) is well below the values observed in hair of other mammalian omnivores (–1.6 to 4.3 ‰), but the mean Δ^15^N value (3.5 ‰) falls within the range (– 0.5 to 4.1 ‰ [30, 34, 35, 37]; Table 3). Our observed value of Δ^15^N is in accordance with the general assumption that the δ^15^N of a consumer changes predictably as trophic level increases and thus is enriched by ~3.4 ‰ relative to diet, mainly due to the excretion of isotopically light nitrogen in urinary waste products [16].

**Table 3:** Average values of trophic discrimination factors for stable isotopes of carbon (Δ^13^C) and nitrogen (Δ^15^N) in other mammalian omnivores.

Study species	n	Δ^13^C	Δ^15^N	Source
Domestic pig (*Sus scrofa*, breed *Seghers*)	5	0.2	2.7	[37]
Rat (*Rattus rattus*)	48	-1.6 to 1.1	-0.5 to 2.5	[30]
Sprague-Dawley rat	24	2 to 4.3	2.3 to 4.1	[34]
Striped skunk (*Mephitis mephitis*)	16	1.2 to 1.6	3.2 to 3.8	[35]

Values of Δ^13^C and Δ^15^N are presented in ‰.

The observed differences between our Δ^13^C value and published values for Δ^13^C in the hair of other mammalian omnivores could be a consequence of different diet types used in those studies. Despite the lacking information on the effects of diet on Δ^13^C values in mammalian hair, previous studies have suggested that diet type can affect the value of Δ^13^C (e.g. [5, 6, 58, 59]). Prior to our feeding experiment, the study animals were fed with a pure C_3_ plant-based diet (mainly consisting of a mixture of wheat, barley, and oat). However, at the start of this experiment, the wild boar were switched to a pure C_4_ plant-based diet (corn grain). The negative value of our Δ^13^C may thus be attributed to relatively low nutritional quality of the C_4_ corn grain given the known deficiencies in essential amino acids in corn [60]. Animals feeding on C_4_ plants with low quality protein may have thus used more carbon from carbohydrates and lipids for tissue synthesis, and as a result the proportion of carbon derived from the corn grain was lower than the amount contained in the bulk diet [60–62]. Murray et al. [62] observed a similar pattern in Δ^13^C values of sheep wool, c. 3 ‰ depletion in ^13^C relative to the pure C_4_ diet. Moreover, it has been shown that hair containing the root (as analyzed in this study) are on average ∼0.6 ‰ more depleted in ^13^C than hair without the root, as the roots contain more lipids. Lipid-rich tissues are known to be depleted in ^13^C relative to lipid-poor tissues [54]. However, plucking hair instead of cutting avoids the loss of recently grown hair and thus retains the most recent isotopic information [18].

Our values of trophic discrimination factors for wild boar hair are different than those reported by Nardoto et al. [37] for hair of domestic pig (*Sus scrofa*, breed “Seghers”). Our value for Δ^13^C is lower by 2.5 ‰ and higher by 0.8 ‰ for Δ^15^N. There could be several explanations for such differences. Firstly, the differing diet compositions; their study individuals were fed with a mixed diet composed of 25% soybean, 65% corn, and 10% vitamin and mineral mix. The observed difference in Δ^13^C and Δ^15^N values is likely a result of different protein quality and amounts of essential amino acids of mixed C3- and C4- plant based diet vs. pure C4 diet as used in the current study [63]. Secondly, thousands of years of domestication has resulted in pronounced differences in anatomy and physiology between domestic pigs and their wild ancestors and hence differences in their metabolic pathways may have caused divergent isotopic routing [64–67]. Thirdly, the age differences between the study animals; in our experiment individuals were 3 year old males whereas in the study of Nardoto et al. [37], only 152 day-old females were used. We believe that the lower value of Δ^15^N found in their study is likely an age effect, since 152 day-old individuals are still growing and will therefore have reduced Δ^15^N [27, 34]. Growing individuals are believed to retain more ^14^N in their body pool as they use more dietary protein for tissue synthesis rather than removing N as waste, which results in lower Δ^15^N values [27, 34]. Lastly, they do not specify the type of hair used in their study and also sampled the hairs used for analysis by clipping (i.e. without root), which may perhaps also contribute to the observed differences in Δ^13^C [54].

We found that the mean values of δ^13^C of the hair samples representing the days 0–9 were significantly different from those representing the days 132–140, whereas the days 34–42 and 132–140 did not differ. There were no differences in the mean δ^15^N values of the hair samples representing the three time periods. These findings suggest that the time required for the δ^13^C value of hair to be stabilized with diet is at least 30 days. This is consistent with previously published study on steers (*Bos taurus*) where the delay between the two isotopic records of diet was estimated to be between 10–20 days [46]. Similarly, Caut et al. [32] reported a 40-day period for hair of shaved rats (*Rattus rattus*) to stabilize with diet. The constant value of δ^15^N observed in our study animals across the duration of the experiment could be caused by a similar δ^15^N signature of both the corn grain and the diet prior to the start of the experiment. On the other hand, this may also suggest that the time for stabilization is shorter for δ^15^N, but this needs further investigation.

Since our feeding experiment was only conducted on four male wild boar of the same age, fed with a single food item, we suggest that further experimental studies should test for possible differences of TDFs (Δ^13^C and Δ^15^N) and hair growth rates when feeding a mixed diet of different nutritional quality. Future studies should also address the range of food resources commonly used by free-ranging wild boar. We have determined the TDFs (Δ^13^C and Δ^15^N) in a single tissue only (hair), and therefore recommend estimating TDFs also for other wild boar tissues with different metabolism. However, TDFs for hair are particularly valuable as they can be sampled non-destructively. In addition, the effect of age and sex on the TDFs values as well as the growth rate of wild boar hair needs to be established (e.g. [34, 58]).

Our estimations of the average hair growth rate and trophic discrimination factors of δ^13^C and δ^15^N in hair of wild boar provides a foundation for interpreting isotopic patterns of free-ranging individuals and can be applied in estimations of problems such as crop damage, particularly in areas with high densities of corn fields, once proportions are established. However, more experimental studies under controlled conditions are needed in order to provide a deeper understanding of the processes underlying the variation in trophic discrimination factors of δ^13^C and δ^15^N. Experiments such as ours provide a stepping-stone for future studies that will focus on wild boar feeding ecology, especially in agriculturally dense areas where wild boar populations continue to increase and expand, and individuals become more habituated to human provided food subsidies, particularly corn.

## Supporting Information

S1 Table Data on lengths (mm) of the shoulder and rump hair of individual wild boar collected at the first sampling period (i.e. after 42 days after the shaving). (PDF)

S2 Table Data on stable carbon isotope ratios (δ^13^C) and stable nitrogen isotope ratios (δ^15^N) of the corn grain and hair samples of individual wild boar. All values reported in ‰.
(PDF)
