# Supplementary material for: Trophic Discrimination Factors of Stable Carbon and Nitrogen Isotopes in Hair of Corn Fed Wild Boar
Source: PLoS One. 2015 Apr 27;10(4):e0125042. doi: 10.1371/journal.pone.0125042 (PMC4411150; doi:10.1371/journal.pone.0125042)
Supplement: S1 Table — (PDF) [file pone.0125042.s001.pdf]

S1 Table. Data on lengths (mm) of the shoulder and rump hair of individual wild boar collected at the first sampling period (i.e. 42 days after the shaving).

Individual	Body location	Length	Individual	Body location	Length	Individual	Body location	Length
ind1	rump	49	ind2	shoulder	42	ind3	shoulder	45
ind1	rump	43	ind2	shoulder	53	ind3	shoulder	48
ind1	rump	38	ind2	shoulder	46	ind3	shoulder	54
ind1	rump	43	ind2	shoulder	45	ind3	shoulder	52
ind1	rump	45	ind2	shoulder	40	ind3	shoulder	47
ind1	rump	40	ind2	shoulder	43	ind3	shoulder	52
ind1	rump	43	ind2	shoulder	53	ind3	shoulder	51
ind1	rump	44	ind2	shoulder	48	ind3	shoulder	48
ind1	rump	45	ind2	shoulder	47	ind3	shoulder	50
ind1	rump	39	ind2	shoulder	42	ind3	shoulder	53
ind1	rump	41	ind2	shoulder	50	ind3	shoulder	53
ind1	rump	42	ind2	shoulder	51	ind3	shoulder	48
ind1	rump	43	ind2	shoulder	45	ind3	shoulder	51
ind1	rump	51	ind2	shoulder	44	ind3	shoulder	50
ind1	rump	43	ind2	shoulder	40	ind3	shoulder	54
ind1	rump	49	ind2	shoulder	40	ind3	shoulder	46
ind1	rump	49	ind2	shoulder	54	ind3	shoulder	53
ind1	rump	48	ind2	shoulder	51	ind3	shoulder	46
ind1	rump	48	ind2	shoulder	47	ind3	shoulder	51
ind1	rump	50	ind2	shoulder	41	ind3	shoulder	50
ind1	rump	43	ind2	shoulder	39	ind3	shoulder	54
ind1	rump	46	ind2	shoulder	43	ind3	shoulder	49
ind1	rump	44	ind2	shoulder	39	ind3	shoulder	50
ind1	rump	40	ind2	shoulder	50	ind3	shoulder	52
ind1	rump	38	ind2	shoulder	51	ind3	shoulder	48
ind1	rump	45	ind2	shoulder	54	ind3	shoulder	51
ind1	rump	47	ind2	shoulder	43	ind3	shoulder	48
ind1	rump	40	ind2	shoulder	41	ind3	shoulder	45
ind1	rump	45	ind2	shoulder	52	ind3	shoulder	48
ind1	rump	42	ind2	shoulder	45	ind3	shoulder	50
ind1	rump	43	ind2	shoulder	39	ind3	shoulder	48
ind1	rump	46	ind2	shoulder	44	ind3	shoulder	56
ind1	rump	43	ind2	shoulder	45	ind3	shoulder	50
ind1	rump	41	ind2	shoulder	47	ind3	shoulder	53
ind1	rump	43	ind2	shoulder	43	ind3	shoulder	48
ind1	rump	40	ind2	shoulder	38	ind3	shoulder	51
ind1	rump	39	ind2	shoulder	44	ind3	shoulder	52
ind1	rump	43	ind2	shoulder	45	ind3	shoulder	47
ind1	rump	40	ind2	shoulder	40	ind3	shoulder	49
ind1	rump	39	ind2	shoulder	44	ind3	shoulder	50
ind1	rump	40	ind2	shoulder	40	ind3	shoulder	45
ind1	rump	43	ind2	shoulder	52	ind3	shoulder	49
ind1	rump	39	ind2	shoulder	39	ind3	shoulder	53
ind1	rump	43	ind2	shoulder	52	ind3	shoulder	52
ind1	rump	42	ind2	shoulder	50	ind3	shoulder	46
ind1	rump	36	ind2	shoulder	44	ind3	shoulder	49
ind1	rump	46	ind2	shoulder	39	ind3	shoulder	47
ind1	rump	42	ind2	shoulder	45	ind3	shoulder	52
ind1	rump	42	ind2	shoulder	52	ind3	shoulder	45
ind1	rump	38	ind2	shoulder	48	ind3	shoulder	60
ind1	shoulder	45	ind2	rump	42	ind3	rump	55
ind1	shoulder	48	ind2	rump	39	ind3	rump	56
ind1	shoulder	45	ind2	rump	49	ind3	rump	52
ind1	shoulder	49	ind2	rump	48	ind3	rump	43
ind1	shoulder	45	ind2	rump	48	ind3	rump	50
ind1	shoulder	43	ind2	rump	46	ind3	rump	46
ind1	shoulder	45	ind2	rump	39	ind3	rump	52
ind1	shoulder	50	ind2	rump	40	ind3	rump	54
ind1	shoulder	46	ind2	rump	50	ind3	rump	55
ind1	shoulder	53	ind2	rump	42	ind3	rump	44
ind1	shoulder	50	ind2	rump	49	ind3	rump	49

Individual	Body location	Length	Individual	Body location	Length	Individual	Body location	Length
ind1	shoulder	49	ind2	rump	43	ind3	rump	50
ind1	shoulder	47	ind2	rump	47	ind3	rump	54
ind1	shoulder	49	ind2	rump	37	ind3	rump	47
ind1	shoulder	50	ind2	rump	43	ind3	rump	45
ind1	shoulder	46	ind2	rump	35	ind3	rump	50
ind1	shoulder	45	ind2	rump	38	ind3	rump	51
ind1	shoulder	50	ind2	rump	40	ind3	rump	49
ind1	shoulder	49	ind2	rump	45	ind3	rump	46
ind1	shoulder	45	ind2	rump	47	ind3	rump	48
ind1	shoulder	46	ind2	rump	42	ind3	rump	53
ind1	shoulder	46	ind2	rump	41	ind3	rump	48
ind1	shoulder	47	ind2	rump	36	ind3	rump	51
ind1	shoulder	49	ind2	rump	43	ind3	rump	52
ind1	shoulder	47	ind2	rump	40	ind3	rump	48
ind1	shoulder	47	ind2	rump	40	ind3	rump	55
ind1	shoulder	48	ind2	rump	38	ind3	rump	43
ind1	shoulder	48	ind2	rump	45	ind3	rump	42
ind1	shoulder	46	ind2	rump	48	ind3	rump	54
ind1	shoulder	52	ind2	rump	47	ind3	rump	46
ind1	shoulder	50	ind2	rump	45	ind3	rump	50
ind1	shoulder	45	ind2	rump	42	ind3	rump	46
ind1	shoulder	52	ind2	rump	44	ind3	rump	52
ind1	shoulder	46	ind2	rump	42	ind3	rump	54
ind1	shoulder	50	ind2	rump	35	ind3	rump	55
ind1	shoulder	47	ind2	rump	38	ind3	rump	49
ind1	shoulder	45	ind2	rump	40	ind3	rump	45
ind1	shoulder	48	ind2	rump	45	ind3	rump	42
ind1	shoulder	50	ind2	rump	37	ind3	rump	44
ind1	shoulder	52	ind2	rump	36	ind3	rump	46
ind1	shoulder	50	ind2	rump	43	ind3	rump	55
ind1	shoulder	44	ind2	rump	44	ind3	rump	48
ind1	shoulder	46	ind2	rump	42	ind3	rump	43
ind1	shoulder	48	ind2	rump	43	ind3	rump	50
ind1	shoulder	50	ind2	rump	40	ind3	rump	57
ind1	shoulder	49	ind2	rump	41	ind3	rump	53
ind1	shoulder	47	ind2	rump	47	ind3	rump	50
ind1	shoulder	46	ind2	rump	40	ind3	rump	49
ind1	shoulder	47	ind2	rump	45	ind3	rump	45
ind1	shoulder	45	ind2	rump	43	ind3	rump	55
ind4	shoulder	47	ind4	shoulder	50	ind4	shoulder	48
ind4	shoulder	49	ind4	shoulder	49	ind4	shoulder	55
ind4	shoulder	46	ind4	shoulder	50	ind4	shoulder	49
ind4	shoulder	47	ind4	rump	50	ind4	rump	47
ind4	shoulder	51	ind4	rump	48	ind4	rump	46
ind4	shoulder	46	ind4	rump	46	ind4	rump	45
ind4	shoulder	47	ind4	rump	51	ind4	rump	47
ind4	shoulder	48	ind4	rump	47	ind4	rump	45
ind4	shoulder	50	ind4	rump	48	ind4	rump	46
ind4	shoulder	46	ind4	rump	46	ind4	rump	46
ind4	shoulder	51	ind4	rump	45	ind4	rump	47
ind4	shoulder	47	ind4	rump	49	ind4	rump	46
ind4	shoulder	46	ind4	rump	49	ind4	rump	45
ind4	shoulder	49						
