# Supplementary material for: Trophic Discrimination Factors of Stable Carbon and Nitrogen Isotopes in Hair of Corn Fed Wild Boar
Source: PLoS One. 2015 Apr 27;10(4):e0125042. doi: 10.1371/journal.pone.0125042 (PMC4411150; doi:10.1371/journal.pone.0125042)
Supplement: S2 Table — All values reported in ‰. (PDF) [file pone.0125042.s002.pdf]

S2 Table. Data on stable carbon isotope ratios ($\delta^{13}C$) and stable nitrogen isotope ratios ($\delta^{15}N$) of the corn grain and hair samples of individual wild boar.

**Hair samples**				
	**Individual**	**Body location**	**$\delta^{15}N$**	**$\delta^{13}C$**
**day 0-9**	ind1	rump	8.1	-18.0
	ind1	shoulder	8.0	-18.6
	ind2	rump	8.2	-18.5
	ind2	shoulder	7.6	-19.5
	ind3	shoulder	7.6	-17.8
	ind3	rump	7.5	-18.8
	ind4	rump	7.6	-19.5
	ind4	shoulder	7.7	-19.9
**day 34-42**	ind1	rump	7.9	-14.1
	ind1	shoulder	7.8	-14.2
	ind2	shoulder	7.6	-14.9
	ind2	rump	7.7	-15.0
	ind3	shoulder	7.7	-14.1
	ind3	rump	7.7	-14.2
	ind4	rump	7.7	-13.8
	ind4	shoulder	7.7	-13.7
**day 132-140**	ind1	rump	8.0	-14.5
	ind1	shoulder	7.8	-14.6
	ind2	rump	7.7	-14.3
	ind2	shoulder	7.5	-14.4
	ind3	rump	7.8	-14.6
	ind3	shoulder	7.6	-14.3
	ind4	rump	7.6	-13.9
	ind4	shoulder	7.6	-14.2
**Corn grain**				
			**$\delta^{15}N$**	**$\delta^{13}C$**
			4.0	-12.0
			4.2	-12.0
			4.2	-12.0
			4.4	-12.1

All values reported in ‰.
